# Genetic influence on serum 25-hydroxyvitamin D concentration in Korean men: a cross-sectional study

**DOI:** 10.1186/s12263-018-0621-7

**Published:** 2018-12-19

**Authors:** Songhwa Choi, Hyeonyoung Ko, Kayoung Lee, Joohon Sung, Yun-Mi Song

**Affiliations:** 1 Department of Family Medicine, Samsung Medical Center, Sungkyunkwan University School of Medicine, Irwon-ro 81, Gangnamgu, Seoul, 06351 135-710 South Korea; 2 Health Screening Center Kangbuk Samsung Hospital, Sungkyunkwan University School of Medicine, Seoul, 03181 South Korea; 3 Department of Family Medicine, College of Medicine, Busan Paik Hospital, Inje University, Busan, 47392 South Korea; 4 Department of Epidemiology, School of Public Health and Institute of Health Environment, Seoul National University, Seoul, 03080 South Korea

**Keywords:** Vitamin D, Genetics, Twin study, Ethnicity, Korea

## Abstract

**Background:**

Hypovitaminosis D is prevalent worldwide. It is more prevalent in Eastern Asia region, including Korea. In addition to various environmental factors that influence serum 25-hydroxyvitamin D (25(OH)D) concentration, genetic influence also plays a significant role based on studies estimating the heritability of 25(OH)D in non-Asian populations. The objective of this study was to determine the genetic influence on serum 25(OH)D concentration in Korean men using the twin and family data.

**Methods:**

A total of 1126 Korean male adult twins and family members from the Healthy Twin Study with serum 25(OH)D measurement were included in this cross-sectional study. Intraclass correlation coefficients (ICCs) and heritability were calculated by mixed linear regression analysis and quantitative genetic analysis after adjusting for environmental and lifestyle factors.

**Results:**

Mean (± standard deviation; SD) of serum 25(OH)D concentration was 15.34 ± 6.18 ng/ml. The prevalence of vitamin D insufficiency was 19.8% and that of vitamin D deficiency was 77.9%. After adjusting for age, the highest ICC (0.61) was observed for monozygotic twin pairs while the lowest ICC (0.31) was found for father-son pairs. Age-adjusted heritability was estimated to be 58%. When physical activity, multivitamin intake and season of blood sampling were further considered, the ICC and heritability did not materially change. In the sensitivity analysis after excluding known multivitamin users, age-adjusted heritability was reduced to 44%.

**Conclusions:**

In our study of Korean male twins and family members, heritability of 25(OH)D was moderately high. This supports the finding that genetic factors have significant influence on vitamin D status.

**Electronic supplementary material:**

The online version of this article (10.1186/s12263-018-0621-7) contains supplementary material, which is available to authorized users.

## Background

Vitamin D is one of the most important nutrients associated with health status. Vitamin D deficiency, also known as hypovitaminosis D, is associated with many clinically important diseases, including osteoporosis, cardiovascular diseases, diabetes mellitus, and cancers [1–5]. Serum 25-hydroxyvitamin D (25(OH)D) is a storage metabolite form of vitamin D. Vitamin D insufficiency is defined when serum 25(OH)D concentration is low (20–29 ng/ml) while vitamin D deficiency is considered when its concentration is very low (< 20 ng/ml) [6]. It has been estimated that around one billion people worldwide have insufficient concentrations of vitamin D [6]. In the USA, the prevalence of 25(OH)D insufficiency (< 30 ng/ml) has increased from 69% during 1988–1994 to 76% during 2001–2006 [7].

The major source of vitamin D is cutaneous synthesis through sunlight exposure. Cholecalciferol (25(OH)D_3_) is a form of vitamin D mainly synthesized in the skin. Its association with ethnicity and degree of skin pigmentation has been well documented in previous reports [8–10]. A study using the National Health and Nutrition Examination Survey (NHANES) III data by Zadshir et al. [10] has revealed that Caucasians have higher mean concentrations of 25(OH)D_3_ than Hispanics or Black people regardless of age group. People who have the darkest color of skin due to high melanin pigmentation have the lowest concentration of mean 25(OH)D_3_. The prevalence of hypovitaminosis D (≤ 28 ng/ml) was 81% in Black males, 56% in Hispanic males, and 34% in White males [10].

The skin of Asians, including that of Koreans, is more pigmented than Caucasians’ skin but less pigmented than Black people. Thus, one could easily hypothesize that Asians would show a somewhat similar rate of vitamin D deficiency to Hispanics. However, a study analyzing the Korean National Health and Nutritional Examination Survey (KNHANES) data revealed that only 13.2% of Korean men and 6.7% of Korean women had sufficient concentrations of serum 25(OH)D (≥ 30 ng/ml) in 2010 [11]. High prevalence of vitamin D insufficiency was also documented in a multicenter study conducted in China, where only 5.4% of all participants had sufficient 25(OH)D concentrations (> 30 ng/ml) in 2013 [12]. In a population-based cohort study in Japan, only 17.5% of the study participants had sufficient concentrations of 25(OH)D (≥ 30 ng/ml) [13]. Such high prevalence of hypovitaminosis D commonly seen in several Eastern Asian countries suggests that some factors other than skin pigmentation might play important roles in determining serum vitamin D concentrations. These factors may include genetic and cultural factors that affect sun exposure and vitamin D intake. Several previous studies have investigated the effect of genetic factors on vitamin D status [8, 14–20]. However, estimated heritability in these studies varied widely from 23 to 80%. In addition, studies on heritability of vitamin D in Asian population are very scarce, with only one study on Chinese adolescent twins [14]. Studies on Asian adults have not been reported yet. Therefore, the objective of the present study was to investigate genetic influence on vitamin D status in Korean male adult twins and their family members by estimating the heritability of serum 25(OH)D concentration considering their relevant lifestyle factors.

## Methods

### Study participants

We selected study subjects from the participants of the Healthy Twin Study. Details on the study design and protocols of the Healthy Twin Study have been described in a previous article [21]. In short, the Healthy Twin Study is a community-based cohort study conducted from April 2005 to April 2014. It recruited the same sex Korean adult twins (≥ 30 years of age) and their first-degree family members from the general population in order to investigate the quantitative loci of complex traits, as well as the role of environment in the etiology of complex diseases, through nationwide advertisement and mailing to the members of the Korean Twin-Family Register. The study participants provided blood samples and completed detailed questionnaires about lifestyle and epidemiologic information. Among 3489 persons who have participated in the Healthy Twin Study at the first time between December 2005 and December 2012, a total of 1131 Korean men were included in the study, after excluding females and participants who were devoid of serum 25(OH)D measurement. Among these participants, we excluded five participants for the following reasons: undetectable serum 25(OH)D concentration (*n* = 4) and inadequate blood sample (*n* = 1). As a result, 1126 participants from 425 families were included in the analyses, which was comprised of 292 monozygotic twin pairs, 68 dizygotic twin pairs, and 766 non-twin individuals (see Additional file 1). Written informed consent was obtained from all participants of this study. Institutional Review Board of each participating center approved the study protocol.

### Measurements

Blood sampling was performed for all participants in the morning (around 10 a.m.) after an overnight fast. Serum was immediately separated and stored at − 70 °C. Serum 25(OH)D concentrations of all study participants were measured by chemiluminescence immunoassay using LIAISON® XL (DiaSorin, Italy) after thawing the frozen serum in a central laboratory. The assay could not discern between D2 and D3 forms of 25(OH)D. Serum concentration of calcium in fresh sera was measured with commercial kit for calcium (colorimetry) on ADVIA 1650 (Siemens, Germany).

Body weight and height were measured twice by trained research assistants according to the standardized protocol. Body mass index (BMI) was calculated by average weight divided by average height squared (kg/m^2^).

Information on smoking status, alcohol consumption, and physical exercise was collected using a self-administered questionnaire. Smoking status was assessed by using two questions: “have you smoked more than 20 packs of cigarette (400 cigarettes) all the while?” and “are you still smoking?”. Then, study participants were categorized into three groups: never smokers, past smokers, and current smokers. Alcohol consumption status was categorized into two groups (current drinker or non-drinker) by the response to the following two questions; “have you ever drunk alcohol?” and “do you still drink alcohol?” Physical exercise was categorized into three groups by the frequency of exercise per week: less than once per week, one to approximately two times per week, and three or more times per week. For categorization of physical exercise, two questions were asked: “do you regularly exercise to the point of sweating?” and “how many times do you exercise per week?” To find out whether the study participant had been taking multivitamins, the following question was used: “how many pills of multivitamin a week do you take?” We defined seasons into either winter or summer based on the month of blood sampling: winter (from November to April) and summer (from May to October).

In the Healthy Twin Study, zygosity was verified by using 16 short tandem repeat markers including sex-determining marker (PekinElmer’s AmpFISTR®, Norwalk, CT) for 67% of twin pairs and a self-administered zygosity questionnaire for 33% of twin pairs. The zygosity questionnaire showed 94.3% accuracy in ascertainment of zygosity compared to that confirmed by the short tandem repeat markers kit [22].

### Statistical analyses

The study participants were categorized according to tertiles of serum 25(OH)D concentration. Characteristics of participants between tertile groups were compared using chi-square test for categorical variables and analysis of covariance for continuous variables with consideration of age. For categorical variables, direct age standardization was done using Korean male population distribution in the year of 2010 as standard population. The trend of serum 25(OH)D was examined according to age distribution using linear regression analysis. Intraclass correlation coefficients (ICCs) between pairs of twins, siblings, and father-son were estimated using mixed linear regression model. Dizygotic twins are genetically similar to non-twin siblings. Therefore, they were included in siblings’ group for statistical analyses. Quantitative genetic analysis [23, 24] was performed to estimate the heritability of serum 25(OH)D concentration (the proportion of total phenotypic variance explained by additive genetic factors). Variance component approach was applied to separate total phenotypic variation (6p^2^) into additive genetic component (6a^2^), unmeasured environmental component (6e^2^), and unmeasured shared environmental components (6c^2^). This model assumes that the effects of environmental factors are common for members of a family while three variance components (6a^2^, 6e^2^, 6c^2^) are independent of each other. Therefore, total phenotypic variance becomes the sum of these three factors (6p^2^ = 6a^2^ + 6e^2^ + 6c^2^). Heritability can be estimated as the proportion of observed phenotypic variance attributed to additive genetic component (6a^2^/6p^2^).

To examine the influence of multivitamin intake on heritability estimation, we performed sensitivity analysis after excluding participants who reported of using multivitamin supplements. We included the participants who did not take multivitamin supplements and those who did not respond to the relevant questionnaire, assuming these people as non-regular users of any nutritional supplements. ICCs and heritability were calculated as described above.

Analyses were conducted using PAWS Statistics (ver. 21.0 for Windows; SPSS, Chicago, Illinois), SAS version 9.3 (SAS Institute Inc., Cary, NC, USA), and Sequential Oligogenic Linkage Analysis Routines (SOLAR) Eclipss version 7.6.4 (Southwest Foundation for Biomedical Research, San Antonio, TX, USA).

## Results

The mean (± standard deviation, SD) serum 25(OH)D concentration was 15.34 ± 6.18 ng/ml. A total of 19.8% of all participants had insufficient concentration of serum 25(OH)D while 77.9% of all participants had deficient concentration of serum 25(OH)D.

**Fig. 1:**
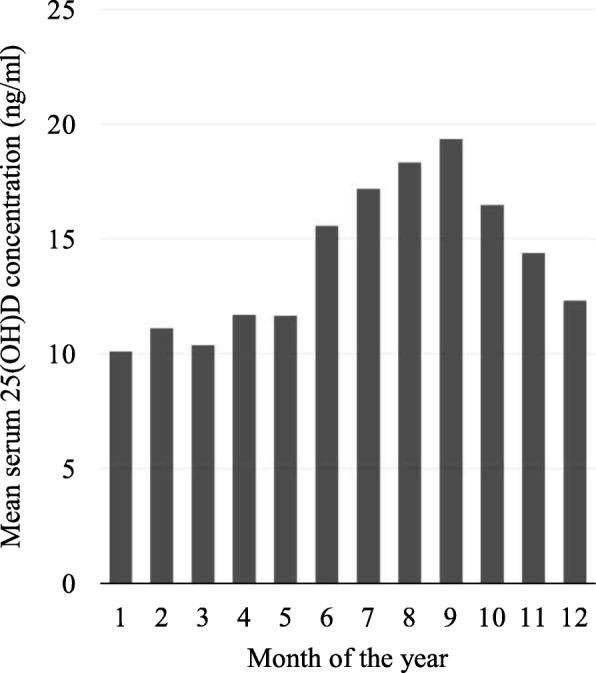
Distribution of mean serum 25-hydroxyvitamin D [25(OH)D] concentration by months of the year [January (= 1) − December (= 12)]

Figure 1 shows how mean serum 25(OH)D concentration is distributed throughout the year according to the time of blood sampling. The highest mean 25(OH)D concentration (19.34 ng/ml) appeared in participants whose blood was drawn in September (late summer season) whereas the lowest concentration (10.10 ng/ml) was seen in participants whose blood was drawn in January (winter season). Mean (± SD) 25(OH)D concentrations in the winter and summer were 12.54 ± 5.26 ng/ml and 16.94 ± 6.10 ng/ml, respectively (*P* < 0.001).

Characteristics of all study participants (*n* = 1126) by tertile distribution of serum 25(OH)D concentration with trend results are shown in Table 1. The mean age was increased significantly from the lowest 25(OH)D tertile group to the highest tertile group. The proportion of multivitamin users tended to be higher among participants with higher serum 25(OH)D concentration (*p* for trend < 0.001). Although there were significant differences in smoking status, alcohol consumption, and the frequency of regular physical exercise between 25(OH)D tertile groups and the least percentage of current smokers or alcohol drinkers was observed in the highest 25(OH)D tertile group, no specific trends were found. Body mass index or serum calcium concentration did not differ significantly between 25(OH)D tertile groups.

**Table 1:** Characteristics of study participants

Variables	25-Hydroxyvitamin D, ng/ml			*P* trend^a^
	≤ 11.7 (*n* = 374)	11.8 ~ 17.2 (*n* = 374)	≥ 17.3 (*n* = 378)	
25-Hydroxyvitamin D, ng/ml	9.07 ± 0.15^b^	14.47 ± 0.14	22.42 ± 0.14	< 0.001
Age, year	40.98 ± 14.23	44.47 ± 13.78	47.21 ± 14.37	< 0.001
Body mass index, kg/m^2^	24.57 ± 0.16	24.65 ± 0.15	24.29 ± 0.15	0.152
Smoking				
Current-smoker	40.34 [181]^c^	43.81 [162]	28.52 [134]	
Past-smoker	20.80 [87]	19.96 [93]	25.90 [132]	< 0.001
Never-smoker	38.86 [105]	36.23 [118]	45.58 [112]	
Current-drinker	77.44 [295]	83.40 [301]	62.87 [291]	< 0.001
Physical exercise				
≥ 3 times/week	20.26 [69]	29.43 [84]	22.42 [106]	
1 ~ 2 times/week	18.97 [59]	13.31 [63]	14.78 [63]	< 0.001
< 1 time/week	60.77 [233]	57.25 [217]	62.80 [198]	
Multivitamin intaker	14.2 [59]	20.4 [93]	21.6 [94]	< 0.001
Calcium, mmol/L	2.37 ± 0.01	2.36 ± 0.01	2.36 ± 0.01	0.072

For some study participants, information was not available for smoking status (*n* = 2), alcohol consumption (*n* = 1), physical exercise (*n* = 34), or multivitamin intake (*n* = 10)

^a^*P* values for trend were obtained by linear-by-linear association of chi-square test for categorical variables or by linear regression analysis for continuous variables

^b^Data were presented as age-adjusted mean ± standard error for continuous variables. Age-adjusted mean values were calculated by analysis of covariance test

^c^Data were presented as number (age-standardized percentage) for categorical variables. *n* in brackets indicates the number of study participants

**Fig. 2:**
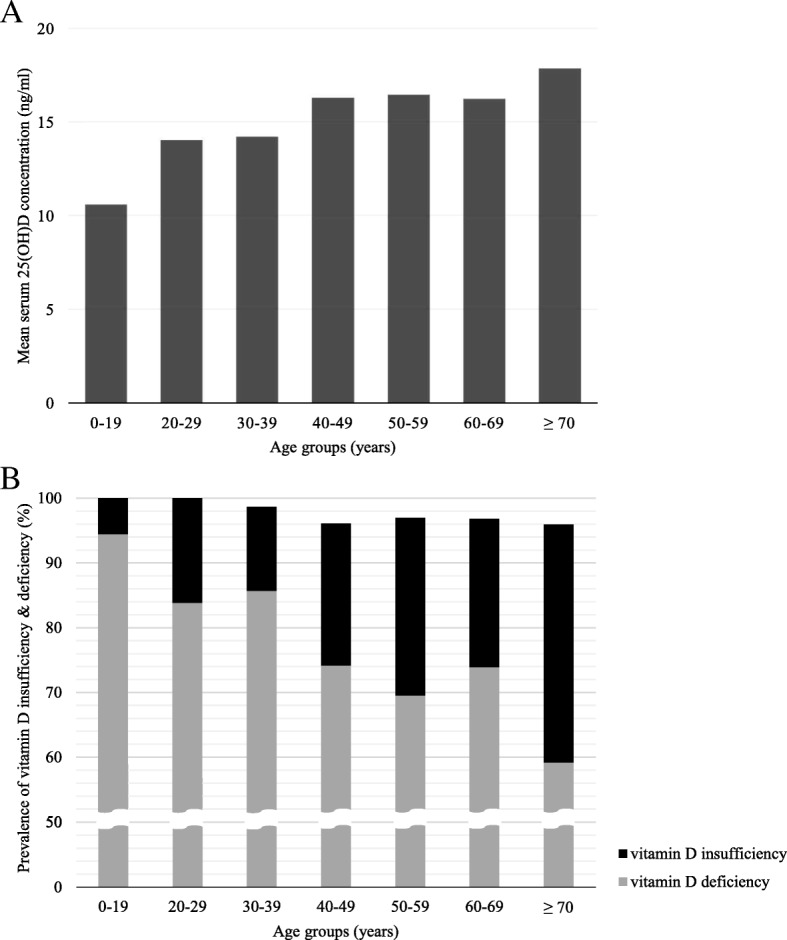
**a** Distribution of mean serum 25-hydroxyvitamin D [25(OH)D] concentration by age groups. When analyzed by linear regression, mean serum 25(OH)D concentration tended to increase with age (*R*^2^ = 0.032, *p* for trend < 0.001). **b** Prevalence of vitamin D insufficiency and deficiency by age groups. As participants’ age increased, the prevalence of vitamin D deficiency tended to decrease. (*p* for trend < 0.001, obtained by linear-by-linear association of chi-square test)

Figure 2 shows distribution of mean serum 25(OH)D concentration and the prevalence of vitamin D insufficiency or deficiency by different age groups. As the age of participant increased, mean serum 25(OH)D concentration tended to increase (*R*^2^ = 0.032, *p* for trend < 0.001) (Fig. 2a). The highest mean concentration of 25(OH)D (17.85 ng/ml) was found in the oldest age group (≥ 70 years) while the lowest mean concentration (10.58 ng/ml) was found in the youngest age group (< 20 years). As can be inferred from this trend of 25(OH)D concentration by age, the prevalence of hypovitaminosis D tended to decrease with increase in age (Fig. 2b).

ICCs between various types of intrafamilial pairs and estimated heritability of serum 25(OH)D concentration are presented in Table 2. Only monozygotic twin pairs showed moderately high correlation: 0.61 in age-adjusted analysis and 0.53 in multivariable-adjusted analysis. Heritability of 25(OH)D was estimated to be moderately high: 58% in age-adjusted analysis and 51% in multivariable-adjusted analysis. Covariates included in these analyses were found to explain about 16% of the total variance. In all models, unmeasured shared environmental components (6c^2^) had negligible influence on phenotypic variance.

**Table 2:** Intraclass correlation coefficients between intrafamilial pairs and heritability of 25-hydroxyvitamin D

	Intraclass correlation coefficients (95% CI)			H^2^ (SE)	VAR (%)
Models	Between MZ twin pairs (*n* = 146 pairs)	Between sibling pairs (*n* = 147 pairs)	Between father-son pairs (*n* = 266 pairs)		
Age-adjusted	0.61 (0.54–0.68)	0.43 (0.34–0.51)	0.31 (0.25–0.38)	0.58 (0.09)	3.7
Model 1^a^	0.54 (0.45–0.61)	0.34 (0.25–0.43)	0.27 (0.20–0.34)	0.52 (0.05)	16.2
Model 2^b^	0.53 (0.44–0.61)	0.34 (0.24–0.43)	0.28 (0.20–0.34)	0.51 (0.09)	16.6

*CI* confidence interval, *MZ* monozygotic, *H*^*2*^ *(SE)* heritability (standard error), *VAR* proportion of variance explained by covariates

^a^In model 1, physical activity, multivitamin intake, and season were additionally adjusted from age-adjusted model

^b^In model 2, smoking status and alcohol consumption were further added to model 1

When we repeated the analysis after excluding the participants who reported the usage of multivitamin supplements, the estimated heritability was reduced to 44% in age-adjusted analysis and 40% in multivariable-adjusted analysis (Table 3).

**Table 3:** Intraclass correlation coefficients between intrafamilial pairs and heritability of 25-hydroxyvitamin D, after exclusion of participants who reported on multivitamin usage

	Intraclass correlation coefficients (95% CI)			H^2^ (SE)	VAR (%)
Models	Between MZ twin pairs (*n* = 98 pairs)	Between sibling pairs (*n* = 115 pairs)	Between father-son pairs (*n* = 167 pairs)		
Age-adjusted	0.57 (0.47–0.66)	0.46 (0.36–0.55)	0.35 (0.27–0.43)	0.44 (0.10)	4.0
Model 1^a^	0.51 (0.40–0.61)	0.37 (0.27–0.47)	0.32 (0.23–0.40)	0.39 (0.10)	15.8
Model 2^b^	0.51 (0.40–0.61)	0.36 (0.26–0.46)	0.32 (0.23–0.40)	0.40 (0.10)	15.5

*CI* confidence interval, *MZ* monozygotic, *H*^*2*^ *(SE)* heritability (standard error), *VAR* proportion of variance explained by covariates

^a^ In model 1, physical activity and season were additionally adjusted from age-adjusted model

^b^ In model 2, smoking status and alcohol consumption were further added to model 1

## Discussion

In these relatively healthy Korean men, the overall prevalence of vitamin D insufficiency and deficiency (serum 25(OH)D < 30 ng/ml) was found to be very high (97.7%). Age, smoking, alcohol consumption, physical exercise, multivitamin intake, and season of blood sampling were identified as factors associated with serum 25(OH)D concentration. The heritability in Korean male twins and their family members was moderately high after considering covariates. To our best knowledge, this is the first study that uses data from twins and family members to determine the heritability of vitamin D status in Eastern Asian adult population. We considered and included covariates known to be associated with serum 25(OH)D concentration in previous studies and made adjustment for these covariates to have more accurate estimation of heritability.

Gender difference in vitamin D status is well known, with women generally showing lower mean serum vitamin D concentration than men [14, 25, 26]. One study done in Chinese adolescents showed gender difference in genetic influence on serum vitamin D, arousing possibility of effect of factors associated with reproductivity on serum vitamin D concentration in females [14]. Moreover, many factors associated with bone metabolism were significantly different in their concentrations and genetic variance according to the menopausal status, probably due to change in estrogen level [15]. In our study, in order to eliminate the differential effect of sex and estrogen in vitamin D status and to focus on determining the genetic influence on vitamin D, we excluded females.

Sun exposure is also known as one of the critical factors that affect serum vitamin D concentration as the main source of vitamin D synthesis [4]. Therefore, latitude may have great influence on vitamin D status [4, 27]. Unfortunately, we did not consider the effect of latitude in our study due to the lack of information. However, we think that the amount of natural sunlight exposure by the effect of latitude is less likely to differ significantly among study participants given that our study participants reside in the area located between a narrow segment of latitude of 35° and 38°. Instead, we included season of blood sampling and physical exercise (which may be relevant to the amount of sun exposure through outdoor activity) as covariates in the analyses. Physical activity was shown to be an important confounding factor in previous studies, although it cannot be considered as a proxy for outdoor sunlight exposure [8, 14]. Seasonal variation in serum 25(OH)D concentration due to change in UV B radiation was clearly observed in this study, consistent with findings from previous studies conducted in various areas around the world [7, 14, 16, 19, 28]. Interestingly, we found that blood samples drawn in September, not in the summer season from June to August, showed the highest mean serum 25(OH)D concentration. This may be attributable to the fact that people go out and do more physical activity when it is not too hot [28].

Aging may lead to decreased synthesis of vitamin D inside the body since 7-decholecalciferol, the precursor of vitamin D_3_, tend to decrease in the skin of the elderly [29]. Moreover, as people get older, they are more likely to stay indoors due to difficulty in movement. Therefore, older people should have lower serum 25(OH)D concentration and higher prevalence of vitamin D deficiency than younger people. Although several studies have shown results supporting this assumption [9, 30–32], controversial findings have been found in different studies, including ours. In our study, we found that mean 25(OH)D concentration increased significantly and the prevalence of vitamin D deficiency decreased as participants’ age increased. Similar findings have been shown in a study of Hovsepian et al., using data from Iranian healthy adults. They compared the vitamin D concentrations among three age groups: 20–39, 40–59, and 60–80 years [33]. Analyses using data from the NHANES of US population have reported inconsistent findings over time. An inverse relation between serum 25(OH)D concentration and age was found in the NHANES of 1988–1994, but not in the NHANES of 2001–2004 [9]. Significant difference in the prevalence of vitamin D deficiency across different age groups also disappeared in the study using data from NHANES of 2005–2006 [34]. Difference in health behavior of the elderly among diverse societies and change of this behavior over time may explain the inconsistency found in the relation between age and vitamin D status. As a society becomes more industrialized and wealthier, the elderly may have more interest in health-pursuing activities, including physical exercise, outdoor activities, and nutritional supplementation such as multivitamin, than younger people. However, we could not examine the validity of this hypothesis in the present study.

Smoking has negative effect on serum vitamin D concentration as shown in many previous studies. Smokers have lower mean serum 25(OH)D concentration and higher prevalence of vitamin D deficiency and insufficiency compared to non-smokers [35–39]. However, in our study, there was no specific relation between smoking and serum 25(OH)D concentration, although the proportion of current smokers was the lowest in the highest 25(OH)D tertile group. This unexpected finding could be related to the behavior of smokers under environmental regulation for smoking in Korea. That is, most smokers go outside to smoke, because indoor smoking is prohibited in Korea. This might have resulted in relative increase in the sunlight exposure of smokers [38].

Alcohol consumption is also known to be inversely associated with vitamin D status [40–42]. In our study, the proportion of current alcohol drinkers was the lowest in the highest 25(OH)D tertile group. However, there was no linear relationship between alcohol intake and 25(OH)D concentration.

In agreement with our study, many studies have shown significant genetic influence on vitamin D concentration in a person, although the estimated heritability ranged widely from 23% to 80% [8, 14–20]. Many genes and proteins involved in the metabolism and actions of vitamin D are known to affect the concentration of serum 25(OH)D, resulting in individual and/or racial differences.

Vitamin D comes into the body or synthesized endogenously and metabolized into its active form and transported in circulation with its binding protein. Genetic alteration may happen in any of these steps, thus possibly altering serum 25(OH)D concentration [43]. From candidate gene studies and genome-wide association studies (GWAS) on vitamin D, several important genes and their single-nucleotide polymorphisms (SNPs) associated with difference in vitamin D status have been discovered, including *CYP2R1*, *CYP27B1*, *CYP24A1*, *GC*, *VDR*, and *NADSYN1/DHCR7* [44, 45]. CYP2R1 and CYP27B1 are cytochrome P450 enzymes known to function in the metabolism of vitamin D by converting vitamin D into its active form, 1α,25(OH)_2_D [46]. Many studies have proven that mutations in these enzymes could alter serum 25(OH)D concentration [17, 43, 47–49]. *GC* gene encodes vitamin D carrier protein, known as DBP. Several SNPs in this gene have also shown significant association with serum 25(OH)D concentration in many studies performed in various ethnicities [8, 43, 50, 51]. Vitamin D receptor (VDR) is a high-affinity receptor for 1α,25(OH)_2_D. It acts as a transcription factor within the cell nucleus [52, 53]. Some genetic changes made in this gene are also associated with variations in serum vitamin D concentration [17, 54, 55], possibly by changing the effectiveness of its transcription action [56]. From a recent large GWAS in European-ancestry participants, two additional loci at *SEC23A* and *AMDHD1* genes were found and overall estimated heritability of serum 25(OH)D concentrations attributable to common genome-wide SNPs was modest (7.5%) [57].

The heritability of serum 25(OH)D concentration tends to differ between different ethnic populations. Wjst et al. have reported that the heritability of 25(OH)D_3_ concentration in their study participants from German families is 80.3% [20]. The heritability of 25(OH)D concentration in Hispanic and African American families has been estimated to be 22.7 to 41.3%, which is lower than that of Caucasians [8]. In Asian subjects, Arguelles et al. have reported that the heritability of 25(OH)D concentration is 68.9% in Chinese adolescent twins [14]. In our study of Korean male adults, the heritability of 25(OH)D concentration was estimated to be 51%, which was lower than that of Caucasians, but higher than that of Hispanics or African Americans. Overall, results from these studies seem to suggest ethnical differences in genetic influence on vitamin D status. Robien et al. have reported that they could not reconfirm the associations of *VDR* or *CYP27B1* genes with vitamin D concentration in Chinese adults living in Singapore, although such association is commonly found in studies conducted in white people [43]. Although the author of this study mentioned that difference in size of study population, latitude, and covariates used in the adjustment might be possible reasons for such racial discrepancy, ethnical differences in genetic regulation of vitamin D cannot be excluded. However, more studies need to be conducted in various populations in order to find out the genetic basis of vitamin D regulation.

Another possible reason for the wide range of heritability reported in previous studies of genetic influence on vitamin D is difference in the season of blood sampling between studies. Karohl et al. have reported seasonal variability in the heritability of vitamin D concentration in US middle-aged White male twins: 70% in the winter time and 0% in the summer time [16]. A study conducted in Swedish adult twins by Snellman et al. has also documented seasonal variation in the heritability of serum 25(OH)D concentration, although the result pointed at an opposite direction: heritability of 48% in the summer and 0% in the winter [19]. In any case, there seems to be a genetically important factor at the stage of cutaneous synthesis that is influenced by the amount of UV B radiation [16, 19]. Further research is needed in this area.

This study has some limitations. First, a cross-sectional study design cannot establish the causal association. Therefore, cautious interpretation for the association between factors and vitamin D concentration in the present study is needed. Second, we only included adult males. Therefore, findings of our study cannot be applied to women, children, or adolescents. Thirdly, we were not able to measure the exact amount of vitamin D intake or outdoor activity, which could result in bias. However, when we conducted sensitivity analysis after excluding multivitamin users, significant heritability persisted with only slight diminution.

## Conclusions

In conclusion, hypovitaminosis D was highly prevalent in Korean men and the estimated heritability of serum 25(OH)D was moderately high. These findings suggest that genetic factors play significant roles in determining the vitamin D status in a person. Further study is warranted to identify specific genes involved.

## Additional file

Additional file 1: Study participants flowchart. (PDF 52 kb)

## Abbreviations

25(OH)D: 25-Hydroxyvitamin D
BMI: Body mass index
CI: Confidence interval
GWAS: Genome-wide association study
ICC: Intraclass correlation coefficient
KNHANES: Korean National Health and Nutritional Examination Survey
NHANES: National Health and Nutrition Examination Survey
SD: Standard deviation
SE: Standard error
SNP: Single-nucleotide polymorphism
