# Supplementary figures and images for: Genetic influence on serum 25-hydroxyvitamin D concentration in Korean men: a cross-sectional study
Source: Genes Nutr. 2018 Dec 19;13:33. doi: 10.1186/s12263-018-0621-7 (PMC6300879; doi:10.1186/s12263-018-0621-7)

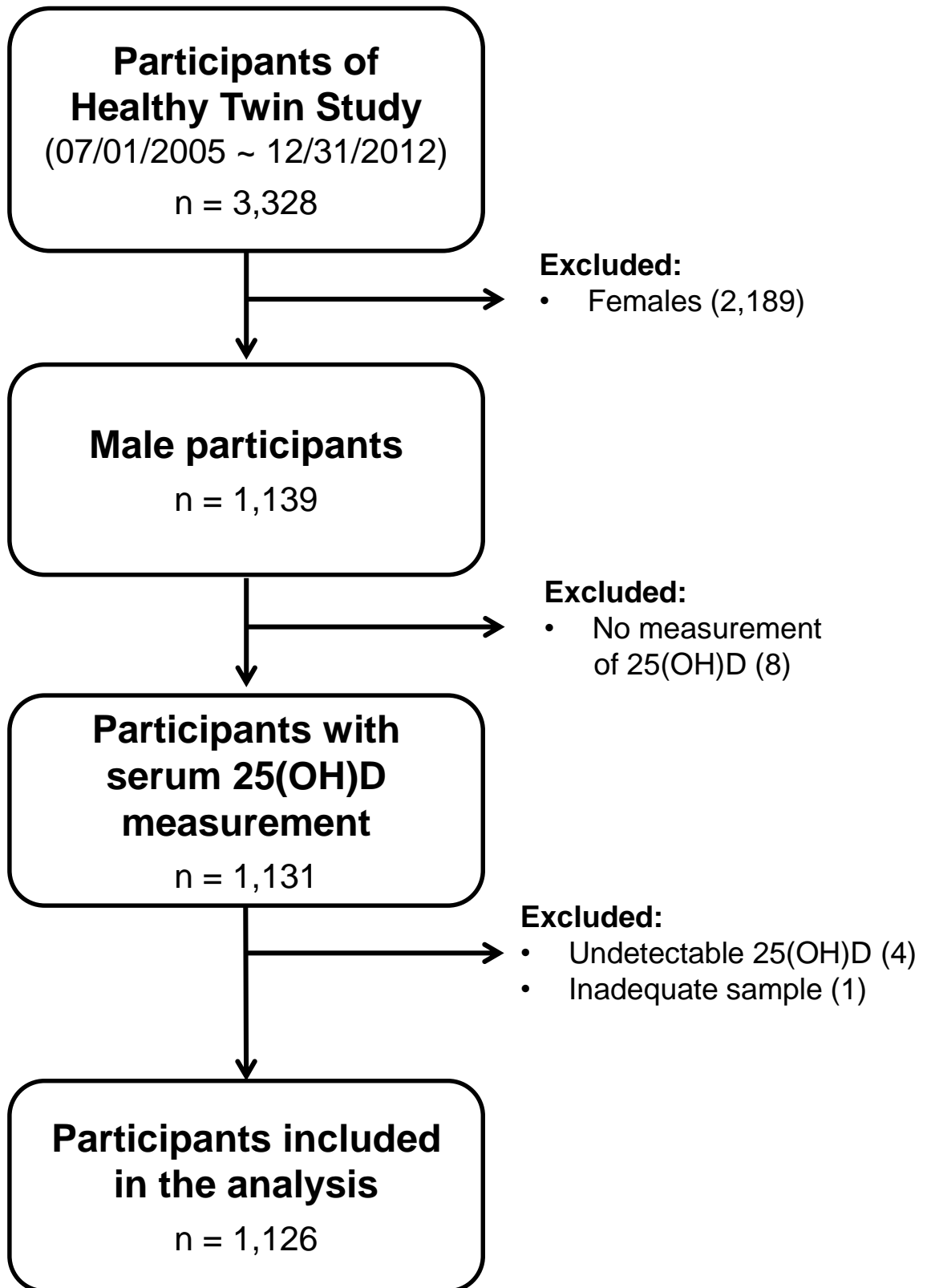

Supplement: Supplementary file 1 — Study participants flowchart. (PDF 52 kb) [file 12263_2018_621_MOESM1_ESM.pdf]
